# Performance evaluation of the Streck ARM-D^®^ Kit, *β*-Lactamase for molecular detection of acquired *β*-lactamase genes

**DOI:** 10.1016/j.jgar.2024.08.004

**Published:** 2024-08-19

**Authors:** Brian B. Yoo, Norihisa Yamamoto, Justina Ilutsik Quintero, Maria Jose Machado, Sarah Sabour, Sara Blosser, Maria Karlsson, James Kamile Rasheed, Allison C. Brown

**Affiliations:** a Division of Healthcare Quality Promotion, National Center for Emerging Zoonotic and Infectious Diseases, Centers for Disease Control and Prevention, Atlanta, GA, USA; b Department of Infection Control and Prevention, Osaka University Graduate School of Medicine, Osaka, Japan; c Chenega Government Mission Solutions, LLC, Chesapeake, VA, USA

**Keywords:** Carbapenemase, Molecular detection, Extended-spectrum *β*-lactamase (esbl), AmpC

## Abstract

**Objectives:**

Despite clinical relevance, commercially available molecular tools for accurate *β*-lactamase detection are limited. In this study, we evaluated the performance of the ARM-D^®^ Kit, *β*-lactamase, a commercially available multiplex PCR assay designed to detect nine *β*-lactamase genes, including the five major plasmid-mediated carbapenemases, ESBL and AmpC genes circulating in the United States.

**Methods:**

A diverse collection of 113 Gram-negative isolates, including 42 with multiple *β*-lactamases genes, was selected from the U.S. Centers for Disease Control and Prevention (CDC) & Food and Drug Administration (FDA) Antimicrobial Resistance Isolate Bank, to represent the most frequently detected bacterial species carrying plasmid-mediated *β*-lactam resistance genes.

**Results:**

Results were compared with whole genome sequence data. Of 164 *β*-lactamase gene targets with 49 unique variants, all were detected correctly without any cross-reactivity. The sensitivity and specificity were 100% (164/164) and 99.9% (852/853), respectively.

**Conclusion:**

The ARM-D^®^ Kit, *β*-lactamase detected a wide range of *β*-lactamase genotypes at a low upfront cost. The Streck assay represents a suitable, comprehensive tool for the detection of key *β*-lactamase resistance genes of public health concern in the United States.

## 1. Introduction

Carbapenemases, extended-spectrum *β*-lactamases (ESBL), and AmpC *β*-lactamases are considered the major “driving forces” of *β*-lactam resistance in Gram-negative bacteria [1]. These mechanisms are often encoded on plasmids, significantly increasing the transmission of antibiotic resistance between patients, animals, and the environment [2]. Time-intensive phenotypic detection methods are available for the clinical/public health laboratory, but few commercial tools are currently available for the molecular detection of carbapenemase, ESBL and AmpC *β*-lactamase genes. Five major carbapenemases genes (*bla*_KPC_, *bla*_NDM_, *bla*_OXA-48-like_, *bla*_IMP_, *bla*_VIM_), have been reported in the United States, with *bla*_KPC_ found most frequently. However, the incidence of other carbapenemases has also increased according to data collected from Antimicrobial Resistance Laboratory Network at the U.S. Centers for Disease Control and Prevention (CDC) [2]. Among ESBLs, though *bla*_TEM_ and *bla*_SHV_ had been dominant until the 1990s, *bla*_CTX–M_ is now the most frequently identified group of ESBLs worldwide [3]. CTX-M-producing organisms have widely disseminated not only in clinical settings but also in the environment and food, and they are also identified as community-acquired pathogens [4]. While the Clinical and Laboratory Standards Institute (CLSI) provides guidance for phenotypic detection of both carbapenemases and ESBLs, there is no standardized phenotypic method to confirm or screen for the presence of AmpC *β*-lactamases [5]. AmpC *β*-lactamases can be constitutively overexpressed by either deregulation of *bla*_AmpC_ genes on a chromosome or acquisition of *bla*_AmpC_ genes on a plasmid [6]. The *bla*_CMY_ is the most predominant family among plasmid-mediated AmpC *β*-lactamases and *bla*_CMY-2_ is the most common variant worldwide [7]. The *bla*_DHA_ represents another plasmid-mediated AmpC-type *β*-lactamase gene that has been globally reported and commonly coexists with other antibiotic resistance genes [8]. The number and diversity of *β*-lactamases are increasing, and *β*-lactamase-producing organisms are found in variable niches such as healthcare settings, environment and food.

Molecular methods to detect *β*-lactamases have several advantages such as rapid turn-around time and definitive identification of specific resistance genes. Several commercial kits have been developed for the detection of *β*-lactamases genes. For instance, the Xpert^®^ Carba-R (Cepheid, Sunnyvale, CA, USA) is one of the platforms approved by U.S. Food and Drug Administration (FDA) that can detect the five major carbapenemase genes [9]. In addition, BD MAX^™^ Check-Points CPO assay (Check-Points, Wageningen, Netherlands), Verigene^®^ BC-GN Nucleic Acid Test (Nanosphere, Inc., Northbrook, IL, USA), BioFire^®^ FilmArray^®^ BCID2 Panel (BioFire Diagnostics, Salt Lake City, UT, USA), cobas^®^ ePlex BCID Panels (GenMark Diagnostics, Pleasanton, CA, USA), Acuitas^®^ AMR Gene Panel (OpGen, Rockville, MD, USA), and ARM-D^®^ Kit, *β*-Lactamase (Streck, La Vista, NE, USA) represent other molecular assays currently available in the United States.

In this evaluation, we assessed the performance of the Streck ARM-D^®^ Kit, *β*-Lactamase (Streck assay), a commercially available research-use-only multiplex polymerase chain reaction (PCR) assay. This assay detects nine relevant groups of *β*-lactamase genes predominantly circulating among Gram-negative bacteria in the United States [10], including carbapenemases (*bla*_KPC_, *bla*_NDM_, *bla*_VIM_, *bla*_IMP_, *bla*_OXA-48-like_), ESBLs (*bla*_CTX–M-15_, *bla*_CTX–M-14_), and AmpCs (*bla*_DHA_, *bla*_CMY-2_). Here, we evaluated the performance of this kit in comparison with whole genome sequencing data.

## 2. Material and methods

### 2.1. Bacterial isolates

We examined the Streck assay using 113 bacterial isolates from the United States Centers for Disease Control and Prevention (CDC) & the United States Food and Drug Administration (FDA) Antimicrobial Resistance Isolate Bank (AR Bank) [11] (Table 1). These isolates were phenotypically and genotypically characterized and contained 164 iterations of 49 different *β*-lactamase genes and variants. All bacterial isolates had been characterized previously by bacterial identification using Matrix Assisted Laser Desorption/Ionization Time of Flight Mass Spectrometry (MALDI ToF-MS) (Bruker Daltonik GmbH, Bremen, Germany) or 16S rRNA and whole genome sequencing (WGS) by either PacBio RS2 (Pacific Biosciences, Menlo Park, CA) or HiSeq 3000 (Illumina, San Diego, CA) platform. This evaluation included 17 species (15 Enterobacterales, *Pseudomonas aeruginosa*, and *Acinetobacter baumannii* complex) exhibiting a range of resistance genes.

### 2.2. Assay procedure

The Streck assay was performed per the manufacturer’s instructions for use (IFU); however, to simplify the process and minimize expenses while maintaining performance, we did not use a commercial DNA extraction kit. Isolates were incubated overnight on Trypticase Soy Agar with 5% Sheep Blood (TSA II) (Thermo Fisher Scientific Inc., Waltham, MA) for 16–18 h at 35±2°C. Bacterial cell lysates were generated by treating with 0.1 M NaOH at 95–99°C for 10 min, followed by centrifugation in Tris–HCl buffered molecular grade water at 2–8°C for 3 min. Lysates were stored at −20°C until use.

Each lysate was evaluated by the Streck assay using the Applied Biosystems 7500 Fast Dx (Thermo Fisher Scientific Inc., Waltham, MA) instrument protocol: Hot Start was performed at 98°C for 30 s, followed by 30 cycles of denaturation (98°C for 10 s), annealing (60°C for 15 s), and detection (72°C for 30 s). For data interpretation, the threshold for normalized reporter (Rn) of multiplex primer-probe Mix 1 (*bla*_CMY-2_, *bla*_CTX–M-14_, *bla*_CTX–M-15_), 2 (*bla*_OXA-48_, *bla*_IMP_, *bla*_VIM_), and 3 (*bla*_DHA_, *bla*_KPC_, *bla*_NDM_) was set at 20,000, 30,000 and 20,000, respectively, to avoid false-positives. A threshold cycle of 10–26 was considered positive.

PCR-positive control mix 1–3 (synthetic DNA templates), negative controls (bacterial lysates of *Escherichia coli* ATCC 25922, *Klebsiella pneumoniae* ATCC 700603, or *Klebsiella pneumoniae* ATCC 1706), and non-template controls were included in each run. Lysates were re-extracted and re-run if the assay’s internal control (targeting the conserved region of 16S rRNA) was negative.

### 2.3. Data analysis

The results were compared to whole genome sequence data. Only the variants which were listed in the IFU were considered for calculation of sensitivity (100 × True Positives / [True Positives + False Negatives]), specificity (100 × True Negatives / [True Negatives + False Positives]), positive predictive value (100 × True Positives / [True Positives + False Positives]), negative predictive value (100 × True Negatives / [True Negatives + False Negatives]), and accuracy (100 × [True Positives + True Negatives] / [True Positive + False Positives + Ture Negatives + False Negatives]) as described in CLSI guideline MM17 and statistical analysis by Prism (ver.7.0c, GraphPad Software, La Jolla, CA).

## 3. Results and discussion

In this study, we evaluated the efficacy of the Streck ARM-D^®^ Kit, *β*-Lactamase, which is claimed to detect the five major carbapenemases (*bla*_KPC_, *bla*_NDM_, *bla*_OXA-48-like_, *bla*_IMP_, *bla*_VIM_), ESBLs (*bla*_CTX–M-14_ group, *bla*_CTX–M-15_ group), and AmpC *β*-lactamases (*bla*_DHA_, *bla*_CMY-2_) genes. The evaluation of this kit is relevant as all of these carbapenemase genes have been reported in the United States. Accurate and timely detection of relevant *β*-lactamase genes is necessary to monitor their spread and guide appropriate infection control practices and treatment regimens.

Streck assay results were compared to whole genome sequencing results. The Streck assay’s sensitivity, specificity, positive predictive value and negative predictive value were 100%, 99.9%, 99.4% and 100%, respectively (Table 2). Testing included 29 isolates that were each positive for two *β*-lactamase genes, and 13 isolates that were each positive for three genes (Table 2). The *β*-lactamase variants expected to be detected by the Streck assay were identified correctly 164/164 times. One *bla*_VIM-7_ was tested but not detected; however, this *bla*_VIM_ variant is not a target of the assay, per the brochure [12] provided by Streck, and therefore was excluded from sensitivity calculations. One isolate produced a false-positive result for *bla*_DHA-1_, thus specificity was calculated as 852/853. All results were concordant among 113 isolates assessed for reproducibility by three independent operators (data not shown). Of note, the whole genome sequencing data of the isolate with the false-positive *bla*_DHA_ result revealed a partial *bla*_DHA-1_ sequence (796/1140 nucleotides). It was not listed in the results of the reference whole genome sequencing because it fell below the set threshold. The kit successfully targeted a sequence that may be located within the partial 70% length region.

This report significantly expands the breadth and depth of previously reported organisms and *β*-lactamase gene targets evaluated using the Streck assay [13]. Key additions in this evaluation include *Acinetobacter baumannii* complex, *Pseudomonas aeruginosa*, expanded evaluation of the *Proteeae* tribe [14] and additional genera and species of the Enterobacterales. It also expanded the evaluation of detecting *bla*_OXA-48-like_, *bla*_VIM_, *bla*_IMP_ and *bla*_DHA_ gene variants (Table 1). The genetic variation and diversity of the *bla*_IMP_ gene family is a well-documented assay design challenge. Compared to other carbapenemase genes, the family of *bla*_IMP_ shows a high degree of diversity, which causes difficulty in designing primers and probes to cover as many variants as possible [15]. For example, the Xpert^®^ Carba-R [16], BD MAX^™^ Check-Points CPO [17], and Acuitas AMR Gene Panel [18] are each limited in their detection of *bla*_IMP_ variants, and were mainly designed to detect the *bla*_IMP-1_ group (e.g., *bla*_IMP-1_, _−3_, _−6_, _−10_, _−25_ and _−30_). In this evaluation, the current Streck assay detected all *bla*_IMP_ variants assessed, including *bla*_IMP-8_, _−13_, _−14_, _−15_, _−18_, _−27_ and _−67_, a variety of *bla*_IMP_ variants which are known to circulate globally as well as in the United States. Despite those advantages, one of the disadvantages of the Streck assay is that it is not designed to directly detect these *β*-lactamase genes within clinical samples. If clinical samples such as rectal swabs (e.g., Carba-R and Check-Points CPO) and positive blood cultures (e.g., Verigene, FilmArray, and ePlex) can be directly tested, the Streck assay could be more valuable for public health purposes. However, there was a recent publication that observed misreporting ESBL from positive blood cultures using those direct blood culture tests with a limitation of overreporting (false positive detection of ESBL) [19]. The Streck assay requires non-automatic procedures; in contrast, fully automated assays such as Verigene BC-GN, FilmArray BCID2, and ePlex BCID which can save labor time and avoid human errors involved in DNA or lysates extraction. Based on the IFUs from each manufacturer, Verigene BC-GN detects the five major carbapenemase resistance genes plus an ESBL resistance gene (*bla*_CTX–M_) and additional *bla*_OXA_ groups including *bla*_OXA-23_, _−40_ and _−58_ from positive blood culture within 2 h. In addition to the five major carbapenemase resistance genes, *bla*_OXA-23_ can be also detected by ePlex BCID in 90 min. Moreover, the FilmArray BCID2 assay detects the five major carbapenemases within an hour. However, automation necessitates considerable investment. The cost per assay is approximately $99 for Verigene BC-GN, $115–135 for FilmArray BCID2, and $135–150 for ePlex BCID [20,21]. Based on cost analysis, the Streck assay is more cost-efficient (~$20 per test) than the kits listed above and can be utilized with several different real-time thermocycler platforms, thereby reducing the investment cost compared to other instruments.

Clinical laboratories are increasingly tasked with providing rapid, inexpensive and accurate lab results. In this evaluation, the Streck assay demonstrated high accuracy (>99% sensitive and specific). To note, there were three limitations to this evaluation. First, the existing AR Bank isolate collection covered only 164 out of 473 gene variants specified in the Streck ARM-D^®^ Kit brochure. However, this collection represents the most commonly detected genes and variants in the United States, drawing from Gram-negative bacterial isolates tested by public health partners for healthcare-associated infection surveillance, outbreak investigations and reference testing. Second, to streamline testing and minimize costs, we employed an extraction method that differed from the Streck assay IFU. These modifications still produced highly sensitive and specific results and would not impact validation of the kit as a laboratory developed test prior to implementing for diagnostic testing. Lastly, our evaluation only assessed the use of the Streck assay with the ABI 7500 Fast Dx instrument, although the IFU allows for implementation with various thermocyclers.

## 4. Conclusion

In conclusion, the Streck assay exhibited excellent performance characteristics, detecting a relatively wide range of *β*-lactamase genes and variants among a diverse group of Gram-negative genera and species. In addition, we found this assay to be favourably competitive in cost compared to several other commercially available PCR kits. Notably, this assay likely has a lower upfront investment for laboratories that currently own a real-time thermocycler. Testing from a crude lysate also contributed to decreasing extraction costs. Together, our findings suggest that the Streck assay represented a suitable, comprehensive tool for detection of key *β*-lactamase resistance genes of public health concern in the United States.

## Figures and Tables

**Table 1:** Distribution of study isolates (*n* = 113), by organism and resistance genotype.

Organism	AR Bank isolate number^a^	Resistance genotype									Total
		CMY-2	CTX-M-15	CTX-M-14	OXA-48	IMP	VIM	DHA	KPC	NDM	
*Acinetobacter baumannii*	0033, 0037, 0063^−^, 0083, 0088	0	0	0	0	0	0	0	0	4	4

Enterobacterales											
*Citrobacter freundii*	0021, 0022, 0116*, 0637, 1282*^+^	5	0	0	0	0	1	0	2	0	8
*Klebsiella aerogenes*	0074, 0161	0	0	0	1	1	0	0	0	0	2
*Enterobacter cloacae complex*	0002, 0032, 0038*, 0154, 0163*, 0501*, 0502, 0607, 0834*, 0838, 1109, 1110, 1152*	0	3	2	0	3	3	1	4	2	18
*E. coli*	0048*, 0069*, 0081, 0085, 0104, 0137*, 0151*, 0162*, 0346*, 0348, 0349*, 0503*, 0595*, 0596*, 0859, 1284*^+^,1285^+^, 1286^+^	10	9	5	1	0	0	1	1	6	33
*Klebsiella oxytoca*	0147, 0837	0	0	0	0	1	0	0	1	0	2
*Klebsiella ozaenae*	0051*, 0096	0	1	0	1	0	0	0	1	0	3
*Klebsiella pneumoniae*	0010, 0034, 0039*, 0042, 0046*, 0066*, 0068*, 0075*, 0076, 0079*, 0080, 0107^−^, 0139*, 0140*, 0347, 0361, 0362, 0504*, 0506*, 0525, 0554*, 0555*, 0556*, 0557*, 0599*, 0603, 0604*, 0606*, 0614*, 0615*, 0850*	5	21	2	13	2	2	1	5	6	57
*Kluyvera ascorbata*	0144	0	0	0	0	0	0	0	1	0	1
*Morganella morganii*	0057*, 0133, 0519	0	1	0	0	0	0	2	1	1	5
*Proteus mirabilis*	0155, 0156, 0159, 1117, 1287^+^	0	0	0	0	2	0	0	2	1	5
*Providencia rettgeri*	0082, 1111, 1113	0	0	0	0	2	0	0	0	1	3
*Providencia stuartii*	0618	0	0	0	0	0	0	0	0	1	1
*Raoultella ornithinolytica*	0134	0	0	0	0	0	0	0	1	0	1
*Salmonella enterica* ser.	0410	0	0	1	0	0	0	0	0	0	1
Infantis											
*Serratia marcescens*	0517*, 0520^−^, 0521	1	1	0	0	0	0	0	1	0	3

*Pseudomonas aeruginosa*	0054, 0090, 0092, 0100, 0103, 0108, 0111, 0239, 0241, 0356, 0439, 1112, 1114, 1115, 1116, 1161, 1288^−+^	0	0	0	0	8	6	0	2	1	17

TOTAL	113	21	36	10	16	19	12	5	22	23	164

a Four-digit number that is used to identify each isolate included in the AR Bank. Some isolates carry multiple *β*-lactamases (*) or do not have *β*-lactamases (^−^); AR-0349 *E. coli* has been removed from the CDC & FDA AR Bank collection and is now available at ATCC (BAA-3170); some isolates (^+^) have not been officially added to the AR Bank yet and their AR Bank numbers are subject to change.

**Table 2:** Performance of Streck ARM-D^®^ Kit, *β*-Lactamase among 113 *β*-lactamase-harboring CDC^1^ & FDA^2^ AR Bank isolates including 42 with multiple mechanisms.

*β*-lactamase gene detected by WGS^a^	Variants detected by WGS	Streck ARM-D^®^ results	
		Positive	Negative
CMY-2 (*n* = 21)	CMY-2, 4, 6, 16, 23, 42, 48, 49, 54, 76, 79, 80, 84, 94	21	92
CTX-M-15 (*n* = 36)	CTX-M-1, 15, 55	36	77
CTX-M-14 (*n* = 10)	CTX-M- 9, 14, 27, 65	10	103
OXA-48-like (*n* = 16)	OXA-48, 181, 232	16	97
IMP (*n* = 19)	IMP-1, 4, 8, 13, 14, 15, 18, 26, 27, 67	19	94
VIM (*n* = 12)	VIM-1, 2, 4, 11, 27	12	101
DHA (*n* = 5)	DHA-1, 2	6^b^	107
KPC (*n* = 22)	KPC-2, 3, 4, 5, 6	22	91
NDM (*n* = 23)	NDM-1, 5, 6	23	90

Dual mechanisms (*n* = 29)			
OXA-48-like/CTX-M-15 (*n* = 9)	OXA-48, CTX-M-15	4	N/A
	OXA-181, CTX-M-15	3	N/A
	OXA-232, CTX-M-15	2	N/A
NDM/CTX-M-15 (*n* = 3)	NDM-1, CTX-M-15	2	N/A
	NDM-7, CTX-M-15	1	N/A
KPC/CTX-M-15 (*n* = 3)	KPC-2, CTX-M-15	2	N/A
	KPC-3, CTX-M-15	1	N/A
CMY/CTX-M-15 (*n* = 3)	CMY-2, CTX-M-55	1	N/A
	CMY-42, CTX-M-15	2	N/A
KPC/NDM (*n* = 1)	KPC-2, NDM-1	1	N/A
NDM/VIM (*n* = 1)	NDM-1, VIM-2	1	N/A
VIM/CTX-M-14 (*n* = 2)	VIM-1, CTX-M-9	2	N/A
VIM/CTX-M-15 (*n* = 1)	VIM-27, CTX-M-15	1	N/A
NDM/CMY (*n* = 1)	NDM-1, CMY-6	1	N/A
CTX-M-14/DHA (*n* = 1)	CTX-M-14, DHA-1	1	N/A
CTX-M-15/DHA (*n* = 1)	CTX-M-15, DHA-1	1	N/A
CMY/CTX-M-14 (*n* = 1)	CMY-54, CTX-M-14	1	N/A
CTX-M-14/CTX-M-15 (*n* = 1)	CTX-M-14, CTX-M-55	1	N/A
KPC/CMY (*n* = 1)	KPC-2, CMY-76, CMY-79	1	N/A

Triple mechanisms (*n* = 13)			
NDM/OXA-48-like/CTX-M-15 (*n* = 4)	NDM-1, OXA-232, CTX-M-15	2	N/A
	NDM-5, OXA-232, CTX-M-15	1	N/A
	NDM-1, OXA-181, CTX-M-15	1	N/A
NDM/CMY/CTX-M-15 (*n* = 4)	NDM-1, CMY-4, CTX-M-15	1	N/A
	NDM-1, CMY-6, CTX-M-15	1	N/A
	NDM-5, CMY-42, CTX-M-15	1	N/A
	NDM-6, CMY-42, CTX-M-15	1	N/A
OXA-48-like/CMY/CTX-M-15 (*n* = 2)	OXA-181, CMY-4, CTX-M-15	2	N/A
KPC/VIM/CMY (*n* = 1)	KPC-3, VIM-2, CMY-49	1	N/A
NDM/CTX-M-15/DHA (*n* = 1)	NDM-1, CTX-M-15, DHA-2	1	N/A
NDM/CTX-M-14/CTX-M-15 (*n* = 1)	NDM-1, CTX-M-14, CTX-M-15	1	N/A

TOTAL		165^b^	852

Performance Characteristics			
Sensitivity	164/164 = 100%		
Specificity	852/853 = 99.9%		
PPV^c^	164/165 = 99.4%		
NPV^d^	852/852 = 100%		
Accuracy^e^	1016/1017 = 99.9%		

1 United States Centers for Disease Control and Prevention.

2 United States Food and Drug Administration.

a The number of isolates that harbour one or more beta-lactamases detected by whole genome sequencing.

b DHA false-positive was found.

c Positive predictive value, PPV = TP / (TP + FP).

d Negative predictive value, NPV = TN / (TN + FN).

e Accuracy = (TP + TN) / (TP + FP + TN + FN).
